# Efficacy and Safety of Human Retinal Progenitor Cells

**DOI:** 10.1167/tvst.5.4.6

**Published:** 2016-07-19

**Authors:** Ma'ayan Semo, Nasrin Haamedi, Lara Stevanato, David Carter, Gary Brooke, Michael Young, Peter Coffey, John Sinden, Sara Patel, Anthony Vugler

**Affiliations:** 1 Department of Ocular Biology and Therapeutics, UCL-Institute of Ophthalmology, London, UK; 2 ReNeuron, Guildford, UK; 3 Massachusetts Eye and Ear, Schepens Eye Research Institute, Boston, MA, USA

**Keywords:** retinal degeneration, nestin, dexamethasone, retinal progenitor cells, human

## Abstract

**Purpose:**

We assessed the long-term efficacy and safety of human retinal progenitor cells (hRPC) using established rodent models.

**Methods:**

Efficacy of hRPC was tested initially in Royal College of Surgeons (RCS) dystrophic rats immunosuppressed with cyclosporine/dexamethasone. Due to adverse effects of dexamethasone, this drug was omitted from a subsequent dose-ranging study, where different hRPC doses were tested for their ability to preserve visual function (measured by optokinetic head tracking) and retinal structure in RCS rats at 3 to 6 months after grafting. Safety of hRPC was assessed by subretinal transplantation into wild type (WT) rats and NIH-III nude mice, with analysis at 3 to 6 and 9 months after grafting, respectively.

**Results:**

The optimal dose of hRPC for preserving visual function/retinal structure in dystrophic rats was 50,000 to 100,000 cells. Human retinal progenitor cells integrated/survived in dystrophic and WT rat retina up to 6 months after grafting and expressed nestin, vimentin, GFAP, and *β*III tubulin. Vision and retinal structure remained normal in WT rats injected with hRPC and there was no evidence of tumors. A comparison between dexamethasone-treated and untreated dystrophic rats at 3 months after grafting revealed an unexpected reduction in the baseline visual acuity of dexamethasone-treated animals.

**Conclusions:**

Human retinal progenitor cells appear safe and efficacious in the preclinical models used here.

**Translational Relevance:**

Human retinal progenitor cells could be deployed during early stages of retinal degeneration or in regions of intact retina, without adverse effects on visual function. The ability of dexamethasone to reduce baseline visual acuity in RCS dystrophic rats has important implications for the interpretation of preclinical and clinical cell transplant studies.

## Introduction

In retinitis pigmentosa, retinal degeneration occurs due to numerous different mutations, mainly in the rod photoreceptors,^1^ but also in the supporting retinal pigment epithelium cells.^2^ Cones die secondarily to rods and the loss of vision in this disease usually progresses from periphery to center, with associate remodeling of downstream visual circuits.^3,4^ At present, there is no treatment for this condition or other diseases where photoreceptors degenerate. However, one promising avenue arising from work in basic research involves the transplantation of retinal progenitor cells to slow progression of the disease or restore visual function.^5–8^

Various sources of cells that can replace photoreceptors are being investigated, including those from fetal, embryonic stem cell, and induced pluripotent stem cell sources.^9–12^ In addition to photoreceptor progenitor cells, other cell types also have shown promise in their ability to slow the rate of retinal degeneration.^13–15^

Human retinal progenitor cells (hRPC) that can be expanded in a progenitor state and then differentiated into retinal cells before or following transplantation are an attractive solution to produce a sufficient volume of donor cells for therapeutic application. These cells are obtained from human fetal eyes at 16 to 18 weeks of gestation; a time at which retinal differentiation has been well defined.^16,17^ It has been shown that hRPC can be expanded for multiple passages in an undifferentiated state and then express photoreceptor markers (opsins) upon differentiation in vitro or following subretinal transplantation.^12,18,19^ Importantly, the expansion phase of hRPC can be extended significantly under low oxygen conditions, generating a scalable cell source suitable for widespread clinical application.^19^

We tested the preclinical safety and efficacy of a hRPC line (GS089), which had been expanded to passage 9 under low oxygen conditions under good manufacturing practice (GMP) conditions. The safety of hRPC was tested in NIH-III nude mice, while the effective dosage range of these cells was examined using the Royal College of Surgeons (RCS) dystrophic rat model. These rats experience a degeneration of rods and cones over the first 3 months of life due to the same genetic defect as a group of patients with retinitis pigmentosa,^2,3,20^ and are a well-established preclinical model for testing the ability of human cells to slow the course of retinal degeneration.^14,15^ Importantly, previous work has shown that a clinically applicable dose can be established by conducting a dose-ranging study in these rats.^15,21^

In addition, we also conducted an experiment in wild type (WT) rats to establish if a single efficacious dose of hRPC would have any adverse effects on normal retinal structure and vision. We reported that hRPC are safe and efficacious in these models and that this cell line holds significant potential to slow the rate of vision loss in retinal degenerative disease. We also reported a rather unexpected role for dexamethasone treatment in accelerating the deterioration of baseline visual function in RCS dystrophic rats, which may be linked to a suppression of microglial/macrophage infiltration of the outer retina.

## Methods

### Isolation and Preparation of hRPC

The hRPC line used in this study (GS089) was isolated from the retinae of a single human fetus at 16 weeks of gestation in compliance with the Declaration of Helsinki. Material was obtained from Advanced Bioscience Resources (Alameda, CA), under the quality and safety guidelines for Food and Drug Administration (FDA) Good Tissue Practice conditions. The isolation and culture of hRPC has been described in detail previously.^12,19,22^ In brief, the neural retina was dissociated with collagenase I and expanded at 37°C under low oxygen conditions (3% O^2^, 5% CO^2^, 100% humidity) on human fibronectin-coated flasks in Ultraculture medium (Lonza, Walkersville, MD) supplemented with 20 ng/mL human epidermal growth factor (EGF; Peprotech, Rocky Hill, NJ), 10 ng/mL human basic fibroblast growth factor (bFGF; Peprotech), and 2 mM L-glutamine (Invitrogen, Carlsbad, CA). Cells were passaged at 80% confluence and reseeded at a density of 2 × 10^4^ cells/cm^2^.

The hRPC batches used for transplantation into rats were produced at ReNeuron's Guildford, UK laboratories from a P8 GMP working cell bank, which was thawed and cultured in human fibronectin-coated T500 flasks at 3% O^2^ as described above. After 72 hours in culture, hRPC were harvested with TrypZean (Sigma-Aldrich Corp., St. Louis, MO), followed by defined trypsin inhibitor (Gibco/Thermo Fisher Scientific, Waltham, MA) and Benzonase (Merck & Co., Rockville, MD). The P9 GS089 hRPC then were formulated at 1 × 10^5^ cells/μL in vehicle (Ca^2+^, Mg^2+^-free HBSS containing 0.5 mM N-acetyl-L-Cysteine [HBSS-NAC]) on the morning of each transplantation day and shipped to UCL-Institute of Ophthalmology (UCL-IOO). This stock solution was either used undiluted for the 2 × 10^5^ (200 K) dosage group (2 μL per rat), or diluted appropriately with vehicle to generate cell suspensions for the 1 × 10^5^ (100 K), 5 × 10^4^ (50 K), and 1 × 10^4^ (10 K) dosage groups. On each surgery day, the cells were counted and assessed for viability upon completion of transplants. This was done using a hemocytometer, where cell viability was determined by calculating the percentage of living cells that exclude trypan blue. Using this method, cell viability was consistently >70% upon completion of transplants.

### Animals

The animals used in this study were either pigmented RCS dystrophic rats, pigmented WT (nondystrophic) Lister Hooded rats, or pigmented Crl:NIH-*Lyst^bg^Foxn1^nu^Btk^xid^* homozygous nude mice (NIH-III). The RCS dystrophic rats were bred in-house at UCL-IOO, while WT rats and NIH-III mice were purchased from a commercial supplier (Charles River Laboratories, Wilmington, MA). All RCS dystrophic rats were 23 to 25 days old on the day of transplantation, an age range that has been used previously to measure human cell-derived visual preservation in this model.^13,23^ All WT rats were 28 to 29 days old and the NIH-III mice were 35 to 42 days old on the day of transplantation, an age that corresponds with the maturation of visual function in rodents.^24,25^ Animals were maintained in a 12-hour light/dark cycle, with food and water available ad libitum. All procedures were performed in accordance with UK Home Office regulations and adhered to the ARVO Statement for the Use of Animals in Ophthalmic and Vision Research.

### Subretinal Transplantation

The procedure used for subretinal transplantation of human cells has been described in detail previously^23,26^ and involved injection of hRPC (2 μL total volume for rats and 1 μL for NIH-III mice) using a 30 to 34 gauge Hamilton needle into the subretinal space of anesthetized animals. Successful subretinal injections resulted in a clear retinal detachment in the dorsal retina and any evidence of inadvertent intravitreal injection or retinal hemorrhage was noted. Experimental animals were injected in counterbalanced batches and injections groups were randomized within individual cages. For a detailed injection method please see additional methods in the supplementary material.

### Single Dose Subretinal Injection with Dexamethasone Treatment

Previous studies have shown poor xenograft survival in the absence of dexamethasone treatment,^23,27–29^ with successful preclinical studies in RCS dystrophic rats using a 2-week course of dexamethasone to control acute inflammatory responses.^14,15^ As such, we first wanted to establish this dexamethasone protocol in our laboratory and test the ability of a single dose of hRPC to preserve vision and retinal structure in dystrophic rats 3 months after grafting.

For this initial experiment, a total of 37 dystrophic rats received either 5 × 10^4^ hRPC in 2 μL (50 K group, *n* = 22) or 2 μL of the HBBS-NAC vehicle (0 K group, *n* = 15). This concentration of cells was chosen because 50,000 human cells have been shown previously to be optimal for preserving visual function in RCS dystrophic rats.^15^ In accordance with the published preclinical protocol of Lu et al.^15^ and McGill et al.,^14^ the rats received an intraperitoneal (IP) injection of dexamethasone at 1.6 mg/kg/day (Organon Pharmaceuticals USA, Inc., Roseland, NJ) every day for 2 weeks following surgery and remained on oral cyclosporine A (210 mg/L) from 2 days before surgery until the end of the study. Visual function was assessed in these animals at 12 weeks after grafting using the optokinetic response (OKR) and photoreceptor preservation was quantified by measuring outer nuclear layer (ONL) thickness in histologic sections following sacrifice at 14 weeks after grafting (*n* = 7 animals from the 50 K group and *n* = 4 animals from the 0 K group).

### Dose Ranging Study without Dexamethasone Treatment

During the single-dose study described above, a significant proportion (∼38%) of animals experienced signs of ill health from 1 week after injection and were removed from the study. As the affected animals came from hRPC- and vehicle-treated groups, this adverse reaction was attributed to the dexamethasone injections and it was deemed ethically unacceptable to repeat this protocol for the dose-ranging study. As such, given that human cells have been reported to survive and preserve vision in RCS dystrophic rats on oral cyclosporine alone,^28–30^ we decided to proceed with a dose-ranging study in the absence of dexamethasone.

A total of 64 dystrophic rats were divided into 5 different treatment groups incorporating vehicle (0 K, *n* = 13) and 4 hRPC doses: 1 × 10^4^ (10 K, *n* = 13), 5 × 10^4^ (50 K, *n* = 13), 1 × 10^5^ (100 K, *n* = 12), and 2 × 10^5^ (200 K, *n* = 13). In addition, to assess any potential adverse impact of hRPC on normal vision and retinal structure, we also included a group of WT rats, which received subretinal injections of either 5 × 10^4^ (50 K, *n* = 15) hRPC or vehicle (0 K, *n* = 15). All animals remained on oral cyclosporine A (210 mg/L) from 2 days before surgery until the end of the study and received a single 2-μL injection into the left eye, with the right eye remaining uninjected. All animals were tested by OKR at 12 weeks and *n* = 5 rats per group were euthanized for histology at 14 weeks after grafting. The remaining animals in each group then were retested for OKR at 26 weeks after grafting before being euthanized for histology.

### Measurement of the OKR

A single observer who was blinded to experimental groups performed all behavioral OKR testing. This was done using an established method that distinguishes visual function emanating from left and right eyes by measuring the total amount of time spent head tracking in the clockwise and anticlockwise direction respectively.^13^ The OKR data presented represent total time spent tracking per eye (over 2 minutes) in response to each of 3 different grating frequencies: Coarse (0.125 cycles/deg [C/D]), intermediate (0.25 C/D), or fine (0.5 C/D). An on-screen timer was used during video analysis, a method validated previously in the laboratory for its ability to detect statistically significant differences in OKR.^23^ For full details of the OKR method used, please see additional methods in the supplementary material.

### Tissue Collection and Immunohistochemistry

Rats were deeply anesthetized with sodium pentobarbital (Euthatal; 800 mg/kg) before perfusion fixation with 60 mL 0.1 M PBS followed by 70 mL of 4% paraformaldehyde (PFA) in 0.1 M PBS. All eyes then were removed and orientated by inclusion of the dorsal eyelid. Tissue was post-fixed in 4% PFA overnight and cryoprotected in 0.1 M PBS containing 30% sucrose (both overnight at 4°C). Eyes then were frozen-embedded in OCT compound (TissueTek) and stored at −80°C before 20 μm sectioning on the cryostat.

For the left (injected) eye of each rat, the sampling procedure was as follows: 4 sections per slide, with 4 series and 1 in 5 between each row in the series in peripheral regions, with every section collected through the optic nerve head (ONH) and reverting to 1 in 5 sampling again once past the ONH. For the right (uninjected) eye of each animal, a more restricted number of slides were taken through the peripheral/central retina (to include the ONH).

Four slides spanning the central retina from 1 series of each left eye were stained using immunohistochemistry (IHC) for anti-human nuclei and anti-TRA-1-85 as described previously^26^ or anti-human–specific nestin (Hs nestin), with 1 slide from the central retina of an adjacent series in each left eye processed in the absence of primary antibodies as a negative control. One central slide (containing the ONH) from the right eye of each animal also was stained for anti-human markers. During this screening procedure the retina was counterstained with DAPI to visualize nuclear layers and an antibody against ML-opsin to visualize host cone morphology. Immunostaining occurred in batches with a slide of pelleted GS089 hRPC in each batch as positive control (pellets prepared from hRPC fixed immediately upon cessation of transplants, cryopreserved, frozen, and sectioned at 14 μm on the cryostat).

Upon location of surviving human cells in one series, adjacent series of sections were used to determine the phenotype of engrafted hRPC. The source and optimal working dilution of each primary antibody are listed in Table 1. When counterstaining human cells with primary antibodies raised in mouse, it sometimes was necessary to use a directly conjugated Alexa Fluor 488 anti-human nestin antibody.

**Table 1:**
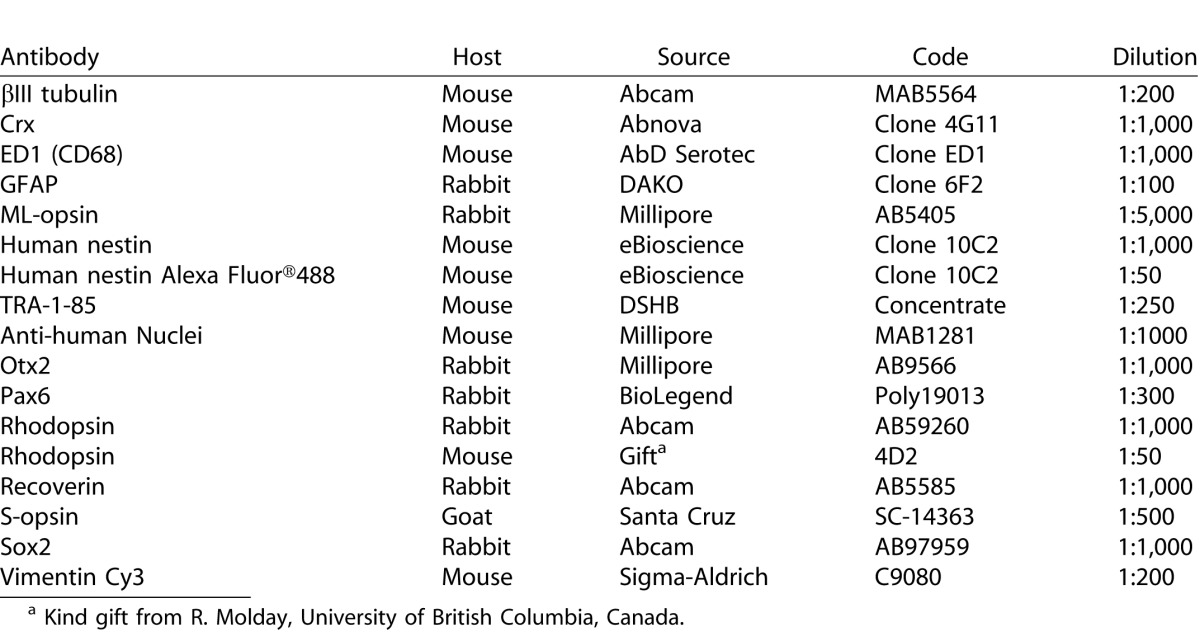
Primary Antibodies, Source, and Working Dilutions.

In addition to the IHC staining detailed above, 1 slide from both eyes of each animal in this study was hematoxylin and eosin (H&E) stained to assess the general health of ocular tissue. This staining and assessment was conducted independently by a UK-based contract research organisation (Sequani Ltd., Ledbury, UK).

### Assessment of Photoreceptor Preservation by Measurement of ONL Thickness

The assessment of ONL preservation was determined for each eye using the series of slides processed for anti-human markers. To visualize the nuclear layers of the retina, images of a standardized area (531.25 × 400 μm) were taken on the 4′,6-diamidino-2-phenylendole (DAPI) counterstain channel using a Leica fluorescence microscope (Leica Camera, Wetzlar, Germany). Slides were coded so that the observer was blinded to the experimental conditions and three images were taken in the dorsal retina of each eye, with the sampling position kept consistent between eyes by always taking photographs in sections where the ONH was visible. Measurements were made at the thickest, most coherent point of the ONL (when present) in each image, with ONL thickness recorded in micrometers, and number of cells. An average thickness then was calculated from the three images of each eye.

### Influence of Dexamethasone on Retinal Microglia/Macrophages

A comparison of the OKR response between animals that had received dexamethasone anti-inflammatory treatment and those that had not revealed a rather striking suppression of visual function emanating from the vehicle-injected (left) and uninjected (right) eyes of treated animals. Given that microglia/macrophages have been shown to persist in the subretinal space of RCS dystrophic rats with preserved ONL/visual function but no detectable human cells,^23^ we hypothesized that these cells may be involved in a potential host retinal response to dexamethasone.

To test this hypothesis, we performed subretinal vehicle injections in the left eye of a small group of RCS dystrophic rats (*n* = 6, 23 days old) and then gave them daily IP injections of either dexamethasone (1.6 mg/kg/day, *n* = 3) or saline (*n* = 3) for 4 days. Animals were monitored closely for any adverse effects and then perfused on the fourth day following surgery. No adverse events were seen during this brief dosing period and all animals appeared in good health before tissue processing.

Both eyes from each animal were frozen and sectioned at 20 μm as described above, with central retinal sections (encompassing the ONH) collected onto slides and processed for IHC to detect microglia/macrophages as follows: endogenous peroxidase activity was reduced by incubating slides for 10 minutes in 0.1 M PBS with 50% methanol and 0.3% H_2_O_2_. Slides then were blocked with 5% normal horse serum in 0.1 M PBS and 0.3% triton X-100 for 1 hour and incubated overnight with the ED1 primary antibody, which detects CD68 expressed in retinal macrophages/microglia.^23^ Sections then were washed with PBS and incubated for 2 hours with biotinylated horse anti-mouse secondary antibody (1:100, Vector Laboratories, Inc., Burlingame, CA). Avidin-biotin signal amplification then was performed for 1 hour using a Vectastain ABC kit (Vector Laboratories) before visualization with a diaminobenzidine chromagen kit (DAKO chromagen kit; DAKO, Carpintiera, CA). Sections were incubated for 5 minutes in this chromagen mixture before stopping the reaction with PBS and cover slipping with glycerol.

Six transmitted light images were taken on a Leica microscope with associated camera and image-grabbing software. These images were spaced equally along the dorsal–ventral axis of the central retinal section, with three sampling points above the ONH: dorsal peripheral (DP), dorsal (D), dorsal central (DC), and three below the ONH: ventral central (VC), ventral (V), and ventral peripheral (VP). From each image, a 277-μm length of retina was analyzed, with a total of 1.7 mm length of retina sampled per eye. Any pigmented tissue adjacent to the retina (e.g., RPE and choroid) was removed and each image was converted to grayscale. Image J (National Institutes of Health, Bethesda, MD) then was used to analyze the grayscale staining profiles, with the threshold for inclusion of staining set at 0 to 112 for all images (where 0 = black). The number of profiles in each image was recorded, with only those profiles above 30 μm^2^ in area included to avoid counting any small fibers/artifacts in the image. In addition to this semiautomated method, we also counted the number of ED1-positive cells in each image manually.

### Safety Study

An independent safety study was conducted at a contract research organization (CRO) according to good laboratory practice (GLP). This study used NIH-III nude mice, an immune-deficient model (lacking natural killer cells/mature T and B cells), which has been used to assess preclinical safety of human cells for ocular application.^15^ Groups of NIH-III mice received a 1 μL subretinal injection into the left eye of 1 × 10^5^ GS089 hRPC (*n* = 45), HBSS-NAC vehicle (*n* = 45), or the Y79 retinoblastoma cell line (positive control, *n* = 46), with all right eyes remaining uninjected. A fourth group of animals (*n* = 45) remained unoperated in both eyes. Cells were prepared at ReNeuron on the morning of transplantation and then shipped to the CRO surgery. The clinical condition, body weight, and food consumption of mice was monitored closely for 9 months (39 weeks) after grafting before euthanization using CO_2_ followed by cervical dislocation. Both eyes and organs (Supplementary Table S1) were examined for macropathology and histopathology following sectioning/H&E staining.

The presence of human cells also was examined in both eyes and other key organs (Supplementary Table S2) of the hRPC-grafted mice by in situ hybridization (ISH) for the human Alu sequence. The presence of this sequence is a reliable indicator of human xenograft survival.^31^ This work was conducted independently under GLP by Histologix Ltd. (Nottingham, UK) and the reagents, staining protocol, and specific conditions used for human Alu-ISH are detailed in Supplementary Tables S3-Supplementary Table S5. A total of 10 sections per eye and 2 sections per organ were stained using the human Alu ISH technique. Positive and negative Alu ISH probes were tested on additional control eyes from WT mice that had an intravitreal injection of hRPC (1μL of 1 × 10^5^ cells/μL), ex-vivo, immediately before fixation/processing for human Alu ISH.

### Statistical Analysis

For OKR data, the total amount of time spent tracking during the two minutes anticlockwise or clockwise was calculated and analyzed statistically using 2-way ANOVA and Bonferonni post hoc tests. To help equalize the variance and normalize the data, before ANOVA analysis, all data were transformed with the equation *Y* = √(*Y*+0.375).^32^ The ONL thickness and number of ED1-positive profiles/cells also were analyzed using 2-way ANOVA followed by Bonferonni post hoc tests. For OKR and ONL, analysis factors were eye (left/right) and cell dose. For the analysis of ED1-stained material, ANOVA factors were eye (left/right) and treatment (dexamethasone or vehicle). All statistical analyses was performed using GraphPad Prism (GraphPad Software, San Diego, CA).

## Results

### hRPC Preserve Vision in Dexamethasone-Treated RCS Dystrophic Rats

**Figure 1:**
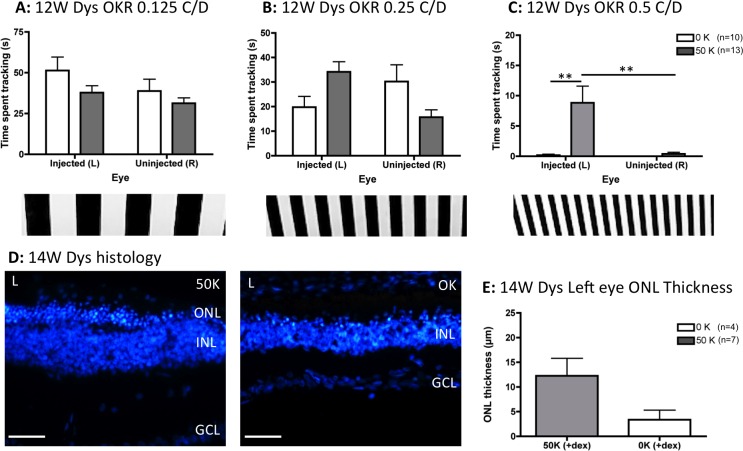
Subretinal injection of a single dose (50 K) of hRPC into dexamethasone-treated RCS dystrophic rats significantly preserves vision 3 months after grafting, as assessed using the OKR. (A) At 0.125 C/D there is no effect of transplant or eye. (B) At 0.25 C/D there is a significant interaction between transplantation and eye but no significance at the post hoc level. (C) At the highest spatial frequency (0.5 C/D), transplantation of hRPC significantly improves vision, with the 50 K (cell transplanted) left eyes tracking significantly better than either vehicle-injected (0 K) left eyes or uninjected right eyes. (D) Representative histology images from the left eyes (L) of cell-injected (50 K) and vehicle-injected (0 K) animals 14 weeks after grafting. (E) Quantification of ONL thickness 14 weeks after grafting reveals more ONL in cell-grafted eyes. However, this does not reach statistical significance. Graphs show the mean ± SEM with *P < 0.001 by Bonferroni's comparison. DAPI staining in *blue*. INL, inner nuclear layer; GCL, ganglion cell layer. *Scale bars*: 50 μm.

After 1 week of dexamethasone treatment approximately 38% of animals (5 vehicle-injected rats [∼33%] and 9 hRPC-injected rats [∼41%]) showed signs of ill health and were removed from the study. Remaining animals were tested for visual function by OKR at 12 weeks after grafting and analyzed by 2-way ANOVA. This showed no significant differences between eye, dose, or any interaction between these factors at the lowest spatial frequency tested (0.125 C/D, Fig. 1A). There was an interaction between eye and dose at the intermediate grating (Fig. 1B, *F*_1,42_ = 7.65, *P* = 0.008) and fine vision was preserved at the highest spatial frequency (0.5 C/D, Fig. 1C), with a significant effect of eye (*F*_1,42_ = 9.49, *P* = 0.004) and dose (*F*_1,42_ = 11.27, *P* = 0.002). Bonferroni post hoc tests confirmed that hRPC injection into the left eye of dexamethasone-treated dystrophic rats significantly improved visual tracking compared to either vehicle-injected left eyes or fellow right eyes (*P* < 0.001, Fig. 1C). Although ONL preservation appeared greatest in hRPC engrafted left eyes (Figs. 1D, 1E), this difference did not reach statistical significance by 2-tailed unpaired *t*-test.

### Detecting a Dose Effect in Dystrophic Rats without Dexamethasone

**Figure 2:**
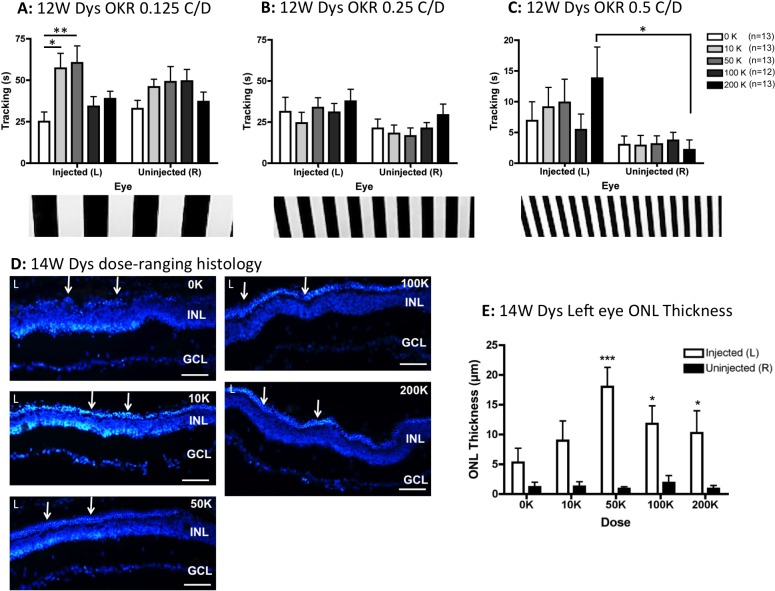
In the absence of dexamethasone treatment, subretinal injection of hRPC into RCS dystrophic rats significantly preserves vision and ONL thickness 3 months after grafting. (A) At 0.125 C/D there is a significant effect of dose, with the 10 and 50 K groups tracking more than the 0 K (vehicle) group at the post hoc level. (B) At 0.25 C/D there is a significant effect of eye but not dose. (C) At 0.5 C/D there also was an effect of eye with a dose effect apparent at the post hoc level, where only the eyes injected with 200 K cells tracked significantly better than the uninjected right eyes. (D) Representative histology images from the left eyes (L) of vehicle-injected (0 K) and cell-injected (10–200 K) animals 14 weeks after grafting show clear ONL preservation (*arrows*) above 50 K. (**E**) The ONL was significantly preserved in left eyes injected with ≥50K cells (compared to uninjected right eyes). Graphs show the mean ± SEM, with *P < 0.05, **P < 0.01, ***P < 0.001 by Bonferroni's comparison. DAPI staining in *blue. Scale bars*: 50 μm.

At 12 weeks after grafting, there was a significant effect of dose at 0.125 C/D (*F*_4,118_ = 3.75, *P* = 0.007), with post hoc comparisons confirming elevated tracking in the left eyes from animals dosed with 10 and 50 K hRPC (relative to left eyes from vehicle-treated rats, *P* < 0.05, as indicated on Fig. 2A). At the intermediate (0.25 C/D, Fig. 2B) and finest (0.5 C/D, Fig. 2C) grating frequencies, eye was the only significant factor (*F*_1,118_ = 5.95, *P* = 0.016 and *F*_1,118_ = 10.13, *P* = 0.002 respectively). At the finest grating, despite a lack of significance between the dosage groups of injected left eyes, a dose effect was found for the highest (200 K) dosage group at the post hoc level by comparing left eyes with fellow right eyes (*P* < 0.05, Fig. 2C). As shown in Figure 2D, there was clear evidence of ONL preservation in the left eyes of dystrophic rats receiving cell injections. Analysis of ONL thickness by 2-way ANOVA confirmed that eye was a significant factor (*F*_1,40_ = 44.00, *P* < 0.0001) but not dose. However, post hoc analysis revealed that hRPC doses of 50 K and above resulted in significantly more ONL preservation in left eyes versus fellow right eyes (*P* < 0.05, Fig. 2E). Although the mean ONL thickness at 12 weeks after grafting was slightly lower in the vehicle-injected eyes of dexamethasone-treated animals (compare Figs. 1E, 2E), this difference did not reach statistical significance.

**Figure 3:**
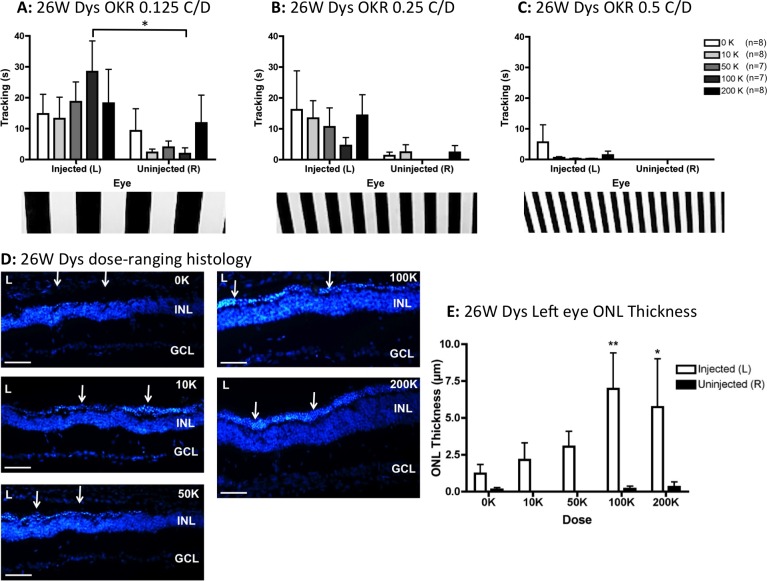
At 6 months after grafting, in the absence of dexamethasone treatment, hRPC injection slows the progressive decline of visual function and ONL thickness in RCS dystrophic rats. (A) Visual function was preserved at the lowest spatial frequency (0.125 C/D), with a significant effect of eye and post hoc significance restricted to eyes injected with 100 K hRPC. (B) At 0.25 C/D, eye remained a significant factor but there was no further significance at the post hoc level. (C) At the finest grating frequency tested (0.5 C/D), dystrophic rats were unable to track with the uninjected right eyes and although some left eye–driven tracking occurred, no significant differences were detectable. (D) Representative histology images from the left eyes (L) of vehicle-injected (0 K) and cell-injected (10–200 K) animals 26 weeks after grafting showing ONL preservation (*arrows*). (E) The ONL was significantly preserved in left eyes injected with ≥100K cells (compared to uninjected right eyes). Graphs show the mean ± SEM, with *P < 0.05 and **P < 0.01 by Bonferroni's comparison. DAPI staining in *blue*, animal numbers in € match those used in (A–C). *Scale bars*: 50 μm.

By 26 weeks after grafting, visual responsiveness of dystrophic rats had diminished in all experimental groups. At 0.125 C/D (Fig. 3A) and 0.25 C/D (Fig. 3B), eye was the only significant factor (*F*_1,66_ = 9.63, *P* = 0.003 and *F*_1,66_ = 14.21, *P* = 0.0004, respectively). However, post hoc tests revealed that only the left eyes treated with 100 K hRPC track better than fellow right eyes at 0.125 C/D (*P* < 0.05, Fig. 3A). Head tracking at 0.5 C/D was minimal from left eyes and completely absent from untreated right eyes at 26 weeks after grafting (Fig. 3C), with no statistically significant differences detected. As shown in Figures 3D to 3E, ONL appeared best preserved in the left eyes of rats injected with a dose of 100 to 200 K hRPC. Eye was a significant factor in ONL preservation (*F*_1,66_ = 17.19, *P* < 0.0001) and although there was no overall effect of dose by 2-way ANOVA, at the post hoc level, 100 to 200 K hRPC resulted in significantly more ONL in left eyes versus fellow uninjected right eyes (*P* < 0.05, Fig. 3E). Interesting, the most significant difference in ONL thickness between left and right eyes occurred in the 100 K dosage group (*P* < 0.001, Fig. 3E), the only group to show a significant difference between left and right eye–mediated tracking (*P* < 0.05, Fig. 3A). Where ONL preservation occurred in dystrophic rats at 26 weeks after injection, this was restricted to the dorsal retina (see low power images in Supplementary Fig. S1).

### Vision and ONL Thickness Remain Stable in hRPC-Injected WT Rats

**Figure 4:**
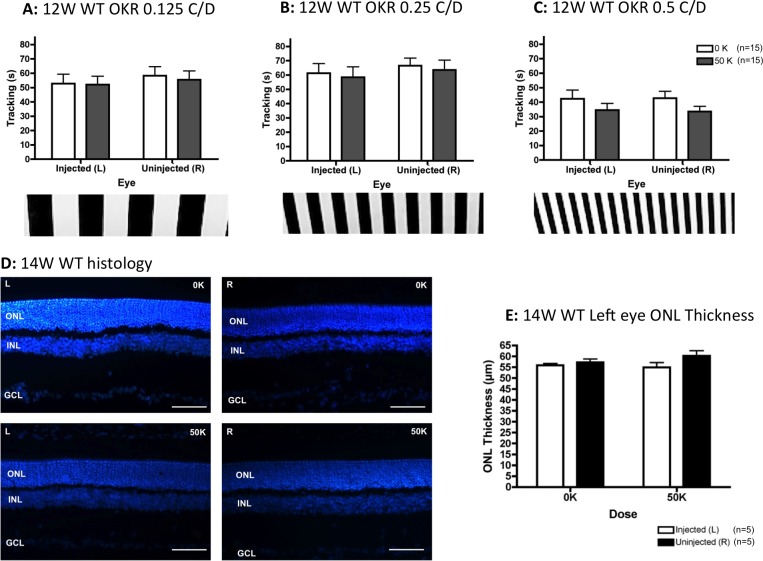
At 3 months after grafting, in the absence of dexamethasone treatment, subretinal injection of hRPC into WT rats had no significant impact on either visual acuity. The rats tracked well at all spatial frequencies tested by OKR: 0.125 C/D (A), 0.25 C/D (B), and 0.125 C/D (C), with time spent tracking the visual stimulus not significantly affected by either hRPC transplantation (0 vs. 50 K) or eye (left versus right). (D) Representative retinal images from the left (L) and right (R) eyes of vehicle-injected (0 K) and hRPC-injected (50 K) animals. (E) There were no statistically significant differences in ONL thickness between either transplant group (0 vs. 50K) or between left and right eyes. Graphs show the mean ± SEM, DAPI-stained nuclei in *blue. Scale bars*: 50 μm.

As part of the dose-ranging study, we also included a group of WT rats receiving subretinal injection of hRPC (50 K) into the left eye in the absence of dexamethasone. We found no significant differences in OKR head tracking or ONL thickness between vehicle and hRPC-injected WT rats at either 12 (Fig. 4) or 26 (Supplementary Fig. S2) weeks after injection.

### HRPC Retain Nestin Expression During Long-Term Survival In Vivo

**Figure 5:**
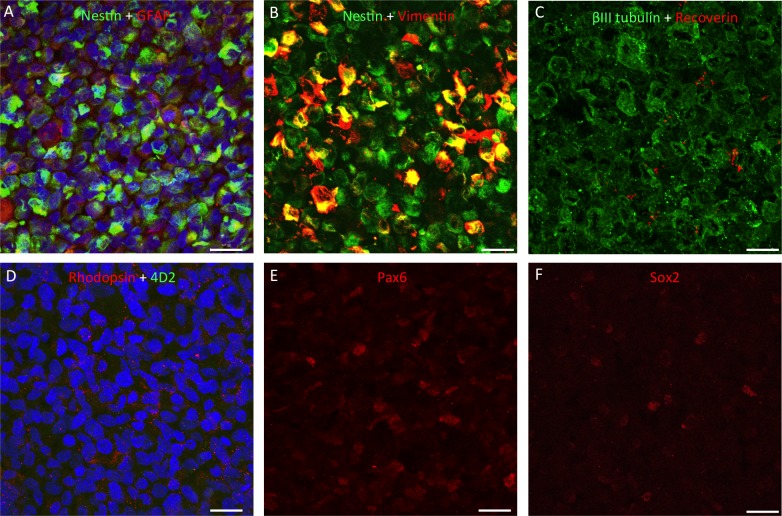
Immunocytochemistry for retinal markers in sections through a pellet of hRPC, fixed immediately upon cessation of surgery. All cells expressed nestin and βIII tubulin, with some positive staining for GFAP and variable levels of vimentin expression (A–C). Human retinal progenitor cells were found to be largely negative for recoverin (*red* in [C]), negative for rhodopsin using the 4D2 antibody (*green* in [D]) but positive for this opsin using a rabbit polyclonal antibody (*red* in [D]). As shown in adjacent sections, subpopulations of hRPC expressed the retinal progenitor markers Pax6 and Sox2 ([E] and [F], respectively). Proteins of interest are color coded and colocalization appears *yellow*. Cell nuclei stained *blue* with DAPI. *Scale bars*: 20 μm.

As shown in Figure 5, on the day of transplantation, hRPC expressed a number of markers consistent with a progenitor cell phenotype. All hRPC expressed nestin, vimentin, and class III β-tubulin (βIII tubulin) to various degrees and some cells also were positive for glial fibrillary acidic protein (GFAP), Pax6, and Sox2. The hRPC were largely negative for the photoreceptor/bipolar cell marker recoverin (Fig. 5C) and also lacked rhodopsin using the 4D2 antibody (Fig. 5D). A positive signal for rhodopsin was detectable using a rabbit polyclonal antibody but this was most likely nonspecific staining due to the lack of colocalization with 4D2. Subpopulations of hRPC were positive for the transcription factors Pax6 and Sox2 (Figs. 5E, 5F) but we were unable to detect Crx, Otx2, ML-opsin, and S-opsin using the antibodies listed in Table 1 (data not shown).

We initially used a mixture of anti-human nuclei and TRA-1-85 to detect surviving human cells; however, the signal provided by this method was very weak, with no definition of cellular morphology. This made it impractical to screen transplanted rat eye sections in this way. Instead, because hRPC express nestin ubiquitously, we used an antibody specific to human nestin to locate grafted cells. Using either method, we were unable to detect any human cells in the left eyes from dexamethasone-treated dystrophic rats (*n* = 7 left eyes sampled at 14 weeks). However, in the dose-ranging study, we found human nestin-positive processes in left eyes from 65% (13 of 20) of hRPC-injected dystrophic rats at 14 weeks and approximately 77% (23 of 30) of the remaining hRPC-injected dystrophic rats at 26 weeks after grafting. The percentage of left eyes containing human processes per group were as follows: 14 weeks (*n* = 5/group) – 10 K (60%), 50 K (60%), 100 K (80%), and 200 K (60%), and 26 weeks (*n* = 5-7/group) – 10 K (∼62%), 50 K (100%), 100 K (∼71%), and 200 K (∼75%). In WT rats we found human nestin-positive processes in 80% (4 of 5 left eyes) of animals sampled at 14 weeks and in 70% (7 of 10 left eyes) of the remaining hRPC-injected rats at 26 weeks. No human cells were detected in any right eyes and, where present in left eyes, hRPC typically spanned 4 to 12 eye sections in one series.

**Figure 6:**
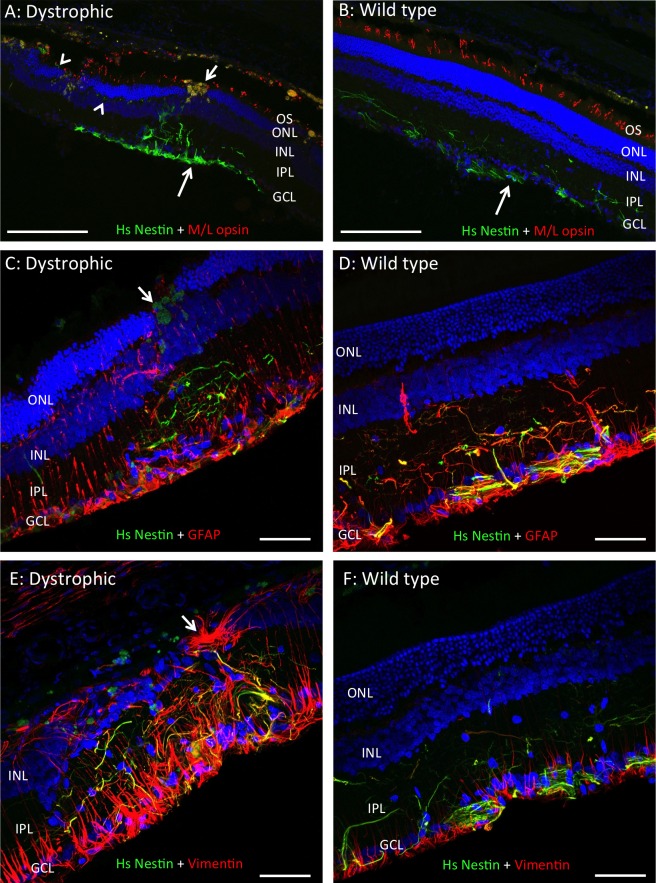
In the absence of dexamethasone, hRPC survive for 6 months following subretinal injection into RCS dystrophic and wild type rats. Representative images from left eyes showing hRPC stained *green* with an antibody to human-specific nestin (Hs Nestin) 6 months after grafting into the RCS dystrophic (A, C, E) and WT (B, D, F) retina. Human cells were located in the inner retina where they extend processes into IPL, with human material also seen at the vitreoretinal interface (*large arrows* in [A] and [B]). Human processes were negative for M/L opsin but positive for GFAP and vimentin to varying degrees (*yellow* colocalization in [C–F]). *Small arrows* in (A) and (C) indicate macrophages in the vicinity of the injection site, while *arrowheads* highlight hRPC processes extending along the outer plexiform layer in (A). Images in (A) and (C) are from rats injected with 200 K hRPC, while all others are from rats injected with 50 K hRPC. *Arrow* in (E) indicates GFAP-positive subretinal scarring. IPL, inner plexiform layer, outer segments (OS). DAPI staining in *blue* and proteins of interest color-coded. *Scale bars*: (A–B) 200 μm, (C–F) 50 μm.

Human nestin-positive processes were consistently negative for rhodopsin, ML-opsin, S-opsin, and recoverin (data not shown). As shown in Figure 6, hRPC retained strong nestin expression up to 6 months (26 weeks) in vivo. Human cells integrated into the host inner retina, with occasional human fibers detectable at the level of the outer plexiform layer (Fig. 6A) but no evidence of human somas within the ONL. Also, we could find no evidence of subretinal masses of hRPC, as reported by Luo et al.^22^ In dystrophic and WT retina, human processes expressed GFAP (Figs. 6C, 6D), vimentin (Figs. 6E, 6F), and βIII tubulin (Supplementary Fig. S3) to varying degrees. A similar pattern of colocalization between these markers and human nestin also was seen in rats killed at 14 weeks after grafting (data not shown). Interestingly, we also found a consistent upregulation of βIII tubulin in the retina of dystrophic rats (Supplementary Fig. S3). As illustrated in Figure 6E and Supplementary Fig. S3A, the presence of hRPC did not always correlate with ONL preservation and large autofluorescent macrophages were seen commonly at apparent injection sites, where ONL was preserved either side of a discontinuity in lamination (arrows in Figs. 6A, 6C). We also found some evidence of subretinal scarring revealed by GFAP staining (arrow in Fig. 6E) and human material along the vitreoretinal interface (large arrows in Fig. 6 and Supplementary Fig. S3).

### Dexamethasone Reduces Baseline Visual Acuity in RCS Dystrophic Rats

**Figure 7:**
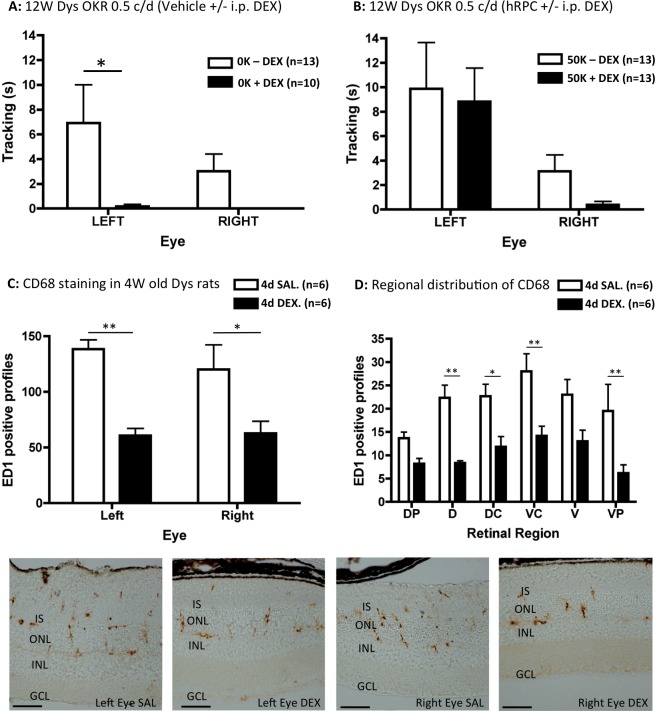
Dexamethasone treatment reduces baseline visual acuity in RCS dystrophic rats. At the highest spatial frequency tested (0.5 C/D), dexamethasone was a significant factor, reducing the amount of time spent head tracking by vehicle (0 K)–injected left eyes (*P* < 0.05) and abolishing the head tracking response from uninjected right eyes (A). In contrast, the level of high spatial frequency head tracking was maintained in the presence of dexamethasone following 50 K hRPC injections into the left eye (B). A brief period of dexamethasone exposure (4 days of IP injection DEX into 23-day-old RCS dystrophic rats) significantly reduced the number of CD68 (ED1)–positive profiles compared to systemic saline injected (SAL) controls in left (vehicle-injected) and right (uninjected) eyes (C). This effect was significant across several regions of dorsal and ventral retina (D). Representative ED1-stained images from left (vehicle-injected) and right (uninjected) eyes are shown below the graphs (all from dorsal retina). ED1-positive staining is *brown*. Graphs show the mean ± SEM with post hoc significance as indicated: *P < 0.05; **P < 0.01. *Scale bars*: 50 μm.

By comparing OKR data from Figures 1C and 2C, it became apparent that there were exceptionally low levels of baseline visual function to the finest grating in dexamethasone-treated animals. When the two 0.5 C/D data sets were compared directly (Figs. 7A, 7B), we found that dexamethasone reduced tracking in response to a sham injection of vehicle (Fig. 7A) but not 50 K hRPC (Fig. 7B). This effect of dexamethasone on the amount of baseline tracking following vehicle injection was significant by 2-way ANOVA (*F*_1,42_ = 7.95, *P* = 0.007), with post hoc analysis confirming a reduction in the left eyes of dexamethasone-treated rats and baseline tracking being completely abolished in the right eyes (*P* < 0.05, Fig. 7A). However, dexamethasone treatment appeared to have no effect on the ability of hRPC to preserve left eye tracking (Fig. 7B), with eye being the only significant factor in this comparison (*F*_1,48_ = 10.44, *P* = 0.002). These data show that hRPC transplantation can preserve fine vision independently of any vehicle-related sham effect. Interestingly, the right eyes of cell-grafted dystrophic rats also produced almost no baseline tracking following dexamethasone treatment (Fig. 7B).

### Dexamethasone Treatment Reduces the Number of Microglia/Macrophages in Both Eyes of Vehicle-Injected RCS Dystrophic Rats

Based on previous observations that ED1 (CD68)–positive microglia/macrophages appear to preserve retinal structure and visual function in RCS dystrophic rats,^23^ we hypothesized that dexamethasone also may exert its effect on baseline visual function through modulation of this cell type. So, given that animals appeared in good health within the first week of dexamethasone treatment, we compared ED1 staining in two groups of young (23 days old) RCS rats in which the left eyes had been vehicle-injected and animals had received 4 daily IP injections of either dexamethasone or saline.

As shown in Figure 7C, using automated analysis, we found a pronounced reduction in the number of ED1-positive cellular profiles in vehicle-injected left eyes and uninjected right eyes of dexamethasone-treated rats. Dexamethasone was a significant factor by 2-way ANOVA (*F*_1,8_ = 25.24, *P* = 0.001), while eye was not, with post hoc tests confirming that dexamethasone treatment reduced ED1-positive profiles in the left and right eyes compared to saline controls (*P* < 0.05, Fig. 7C). Because there were no significant differences between left and right eye, data from both eyes were amalgamated for a regional analysis (Fig. 7D). This showed that dexamethasone (*F*_1,60_ = 49.13, *P* = <0.0001) and retinal region (*F*_5,60_ = 3.50, *P* = 0.008) were significant factors, with a reduction in ED1-positive profiles across the dorsal-ventral extent of retina (*P* < 0.05, Fig. 7D). An independent manual count of ED1-positive cells gave similar results, with dexamethasone treatment and retinal region being significant factors (*F*_1,60_ = 20.57, *P* = <0.0001 and *F*_5,60_ = 5.58, *P* = 0.008, respectively, data not shown). Representative images of ED1 staining from left and right eyes of both treatment groups are shown at the bottom of Figure 7. These findings suggested that dexamethasone works by reducing the migration of microglia/macrophages towards actively degenerating rods/cones in the outer retina.

### Safety Assessments

In the rat eyes examined above by DAPI/H&E (left and right eyes from 104 animals), we could find no evidence of uncontrolled cell growth, tumor formation or unexpected alterations to retinal structure. Furthermore, in an independently conducted GLP tumorigenicity study in NIH-III mice, there was no effect of hRPC injection on body weight gain, food consumption, mortality, or clinical observations of the eyes up to 9 months after grafting. There also were no hRPC-related neoplastic or nonneoplastic changes detected in any eyes or organs. This was not the case in the Y79-positive control group, where mice developed neoplastic changes (i.e., retinoblastoma) in the left eye, thereby confirming the validity of this model for tumorigenicity assessments.

Further investigation of the eyes/organs from NIH-III mice receiving hRPC injections into the left eye found no evidence of human cells in the organs or right eyes. This was with the exception of a single Alu-positive cell found in a right eye section from 1 female animal (data not shown). At 9 months after grafting, human Alu-positive cells were found in the left eyes of approximately 33% of mice (15 of 45). Supplementary Fig. S4 shows the specificity of the Alu-positive probe used in this study. The number of surviving human cells per left eye ranged from 1 to75 (mean, 9.5 ± 5 SEM) and they were found within the inner retina (Supplementary Fig. S4C).

## Discussion

We showed that the hRPC line GS089 is safe and effective in reducing the rate of retinal degeneration and visual deterioration in RCS dystrophic rats. This efficacy result agrees with a recent study, which examined a different hRPC line (GS086) 3 months after grafting into the RCS dystrophic rat model.^22^ Like the study by Luo et al.^22^ (which used dexamethasone), we were also able to detect a clear preservation of fine vision at 3 months after grafting in dexamethasone-treated rats. However, this occurred in the absence of detectable human cells in the present study, a finding that agrees with previous reports of visual preservation in RCS rats in the absence of surviving human iPSC-RPE cells.^23^

For ethical reasons, our dose-ranging study was performed in the absence of dexamethasone treatment. This study established that only 10,000 to 50,000 hRPC were required to preserve visual responses to the broadest grating at 3 months after grafting, with 50,000 cells proving sufficient to preserve ONL thickness at the same time-point. However, we found that higher doses of hRPC (>100,000) were required to preserve fine vision at 3 months after grafting and visual function/ONL thickness at 6 months after grafting. These findings are comparable to those from the only other published preclinical dose-ranging study in RCS rats, which found that preservation of visual response could occur using just 5000 human ES-derived RPE cells and appeared optimal at 50,000 cells.^15^

The disparity between OKR and ONL data at 3 months after grafting in the dose-ranging study may be due to several factors. Firstly, a lack of significance in tracking from the 100 K hRPC-treated eyes may be due to the higher variance in OKR data and baseline tracking caused by dexamethasone omission. Also, other studies have shown that ONL preservation does not always correlate with preserved retinal function.^33,34^ Secondly, the disparity between poor ONL preservation and good levels of tracking to the lowest spatial frequency in left eyes from the 10 K dosage group at 3 months may simply reflect a lack of sensitivity of our histologic sampling method. However, in support of this method, the OKR and ONL data are well correlated for left eyes in the 100 K dosage group at 6 months after grafting.

### Dexamethasone Treatment Reduces Baseline Visual Acuity and Microglia/Macrophages

An unexpected finding in the present study was the effect of dexamethasone treatment on baseline visual acuity in RCS dystrophic rats, with dexamethasone abolishing visual responses to the finest spatial frequency in sham-injected animals. This effect is the opposite of that seen following intravitreal injections of the proinflammatory cytokine IL-1β, which preserves visual acuity and ONL thickness in RCS dystrophic rats.^35^ As the dexamethasone dose used in the present study is likely to exert anti-inflammatory effects, our new findings are consistent with an important role for the inflammatory response in preserving vision during retinal degeneration. This also is consistent with the protective role proposed for microglia in the early stages of neurodegeneration in other diseases of the central nervous system.^36^

The effect of dexamethasone presumably would have made it easier to detect any cell-based preservation of vision in the other RCS rat studies using this drug.^14,15,22^ This is because the measure used to determine optokinetic visual acuity in these studies involves measuring the maximum spatial frequency a rat can detect, rather than the total time spent tracking at different grating frequencies. Interestingly, although the preclinical work for the recent human ES-RPE trial involved dexamethasone, anti-inflammatory treatment was not noted in the clinical protocol.^21^ As such, the initial efficacy obtained from human ES-RPE grafting in patients with advanced AMD would presumably include a significant sham-derived component. However, longer-term follow up of these patients indicates the expansion of pigmented cells and a sustained improvement in visual acuity.^37^

We found that dexamethasone administration significantly reduces the number of ED1 (CD68)–positive microglia/macrophages in the RCS dystrophic retina. These cells previously have been shown to phagocytose photoreceptor debris in the subretinal space of RCS dystrophic rats, where they also have been implicated in the preservation of retinal structure/function in the absence of grafted human cells.^23^ Dexamethasone also has been shown to reduce the phagocytic capacity of macrophages^38–40^ and the trophic response of microglia to brain injury.^41^ By reducing the number of macrophages/microglia, dexamethasone treatment also would be reducing a potential supply of trophic factors,^42,43^ which otherwise could enhance vision through preservation of photoreceptor structure.^13^ However, within the limitations of the present study, we were unable to find statistical evidence for a dexamethasone-mediated acceleration of ONL loss. An alternative explanation to explain how microglia may normally act to enhance baseline visual function in RCS rats could involve some kind of neurotrophin-mediated enhancement of retinal acuity circuits.^44–46^

### Intermediate Filament Expression Is Retained by Xenografted hRPC

The expression profile of hRPC before grafting was consistent with that previously reported for human retinal progenitor cells.^11,12,16,18^ In addition to βIII tubulin and the intermediate filaments nestin and vimentin, we also were able to detect the expression of the intermediate filament GFAP in some hRPC before grafting. This is consistent with previous reports of GFAP expression in cultured hRPC^11,18^ and of nestin, βIII tubulin, and GFAP colocalization in human neural progenitor cells.^47^

Following subretinal transplantation into RCS dystrophic and normal WT rats, hRPC integrated into the inner retina and survived for up to 6 months after grafting in the absence of dexamethasone treatment. This is in line with previous reports of neural progenitor cell integration into the rodent retina in the absence of this drug.^26,30^ As described for hRPC previously,^22,48^ GS089 cells retained expression of nestin in vivo. However, these hRPC also expressed vimentin, GFAP, and βIII tubulin to varying degrees. This is an interesting and important observation, with two potential interpretations: 1, the hRPC remain in their undifferentiated progenitor-like state, or 2, they have differentiated along the glial lineage.

In support of the second possibility, it has been shown that retinal Muller cells express nestin and vimentin in human foetal retina at 15 to 19 weeks of gestation and that hRPC can preferentially differentiate towards a glial fate during neurosphere differentiation experiments.^49^

The morphology of the integrated hRPC and their expression of nestin, vimentin, and GFAP certainly is consistent with a glial phenotype, as none of these markers would be expected in differentiated neurons. However, because we were using nestin to locate human cells it remains possible that a different subpopulation of hRPC differentiated along neural/photoreceptor lineages, which were undetectable in the current experiment. In contrast to a previous study in rhodopsin knockout mice, we were unable to find any evidence of opsin expression by the engrafted hRPC. This discrepancy also could be due to differences between the hRPC line, animal models or opsin antibodies used in the two studies. However, in a recent study looking at another hRPC line (GS086), the human cells survived for 3 months after injection into RCS rats, integrated into the retina, maintained vision by OKR, and expressed nestin.^22^ An absence of hRPC integration into the ONL and lack of photoreceptor marker expression by hRPC in the study of Luo et al.^22^ and our current study strongly suggests that hRPC-mediated preservation of vision in RCS rats is mediated by trophic support, which either preserves photoreceptor structure or increases visual acuity somehow from existing retinal circuitry.^46^

### Conclusions

In conclusion, the GS089 hRPC line appears safe and preserves retinal structure/vision at an optimal dose of 50,000 to 100,000 cells following subretinal injection into a widely used preclinical model of degenerative retinal disease. Although the exact mechanism of hRPC-derived neuroprotection is unclear at present, it may involve either direct neurotrophic release from the human cells or their ability to attract and/or retain neuroprotective macrophages. Importantly, we also showed that hRPC are capable of integrating into the normal WT retina and that their presence does not adversely affect retinal structure or visual function. This is encouraging for the deployment of hRPC in less affected regions of the retina at early stages of retinal disease or even in normal regions of peripheral retina, where they potentially could be used for the sustained intraocular delivery of therapeutic molecules.

## Supplementary Material

Supplement 1

Supplement 2

Supplement 3

Supplement 4

Supplement 5

Supplement 6
