# Supplementary figures and images for: Efficacy and Safety of Human Retinal Progenitor Cells
Source: Transl Vis Sci Technol. 2016 Jul 19;5(4):6. doi: 10.1167/tvst.5.4.6 (PMC4959814; doi:10.1167/tvst.5.4.6)

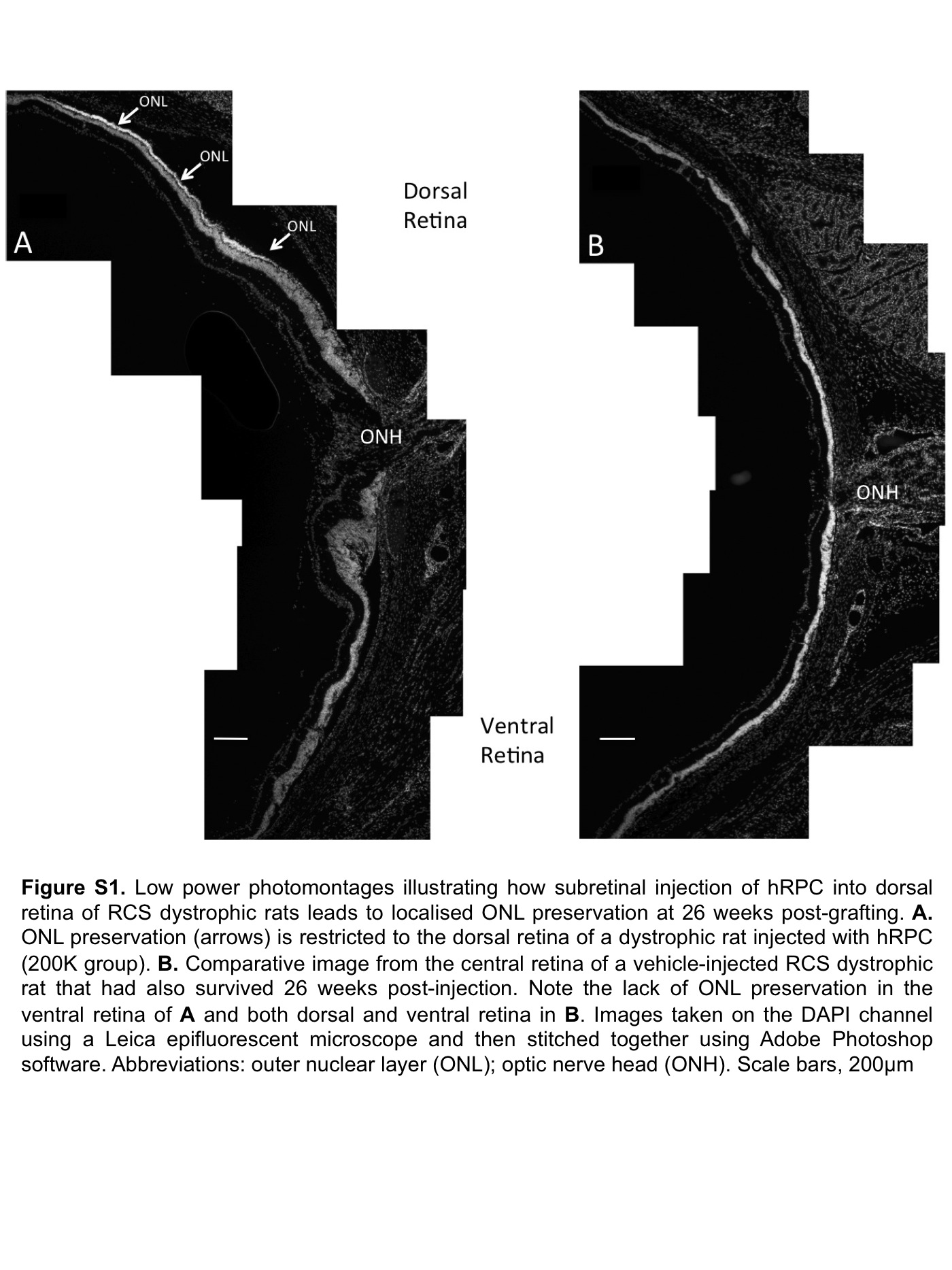

Supplement: Supplement 1 [file i2164-2591-5-4-6-s01.jpg]

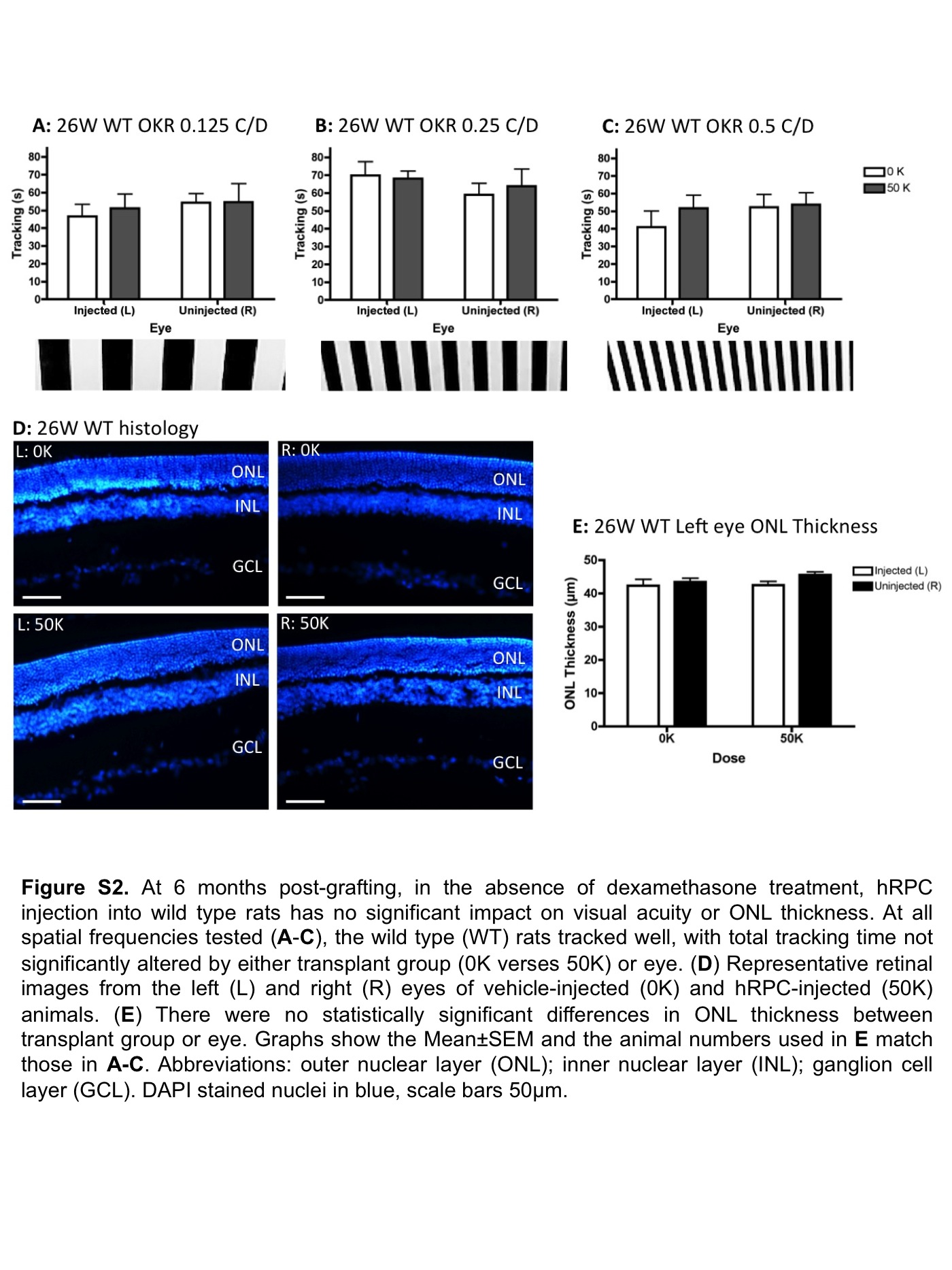

Supplement: Supplement 2 [file i2164-2591-5-4-6-s02.jpg]

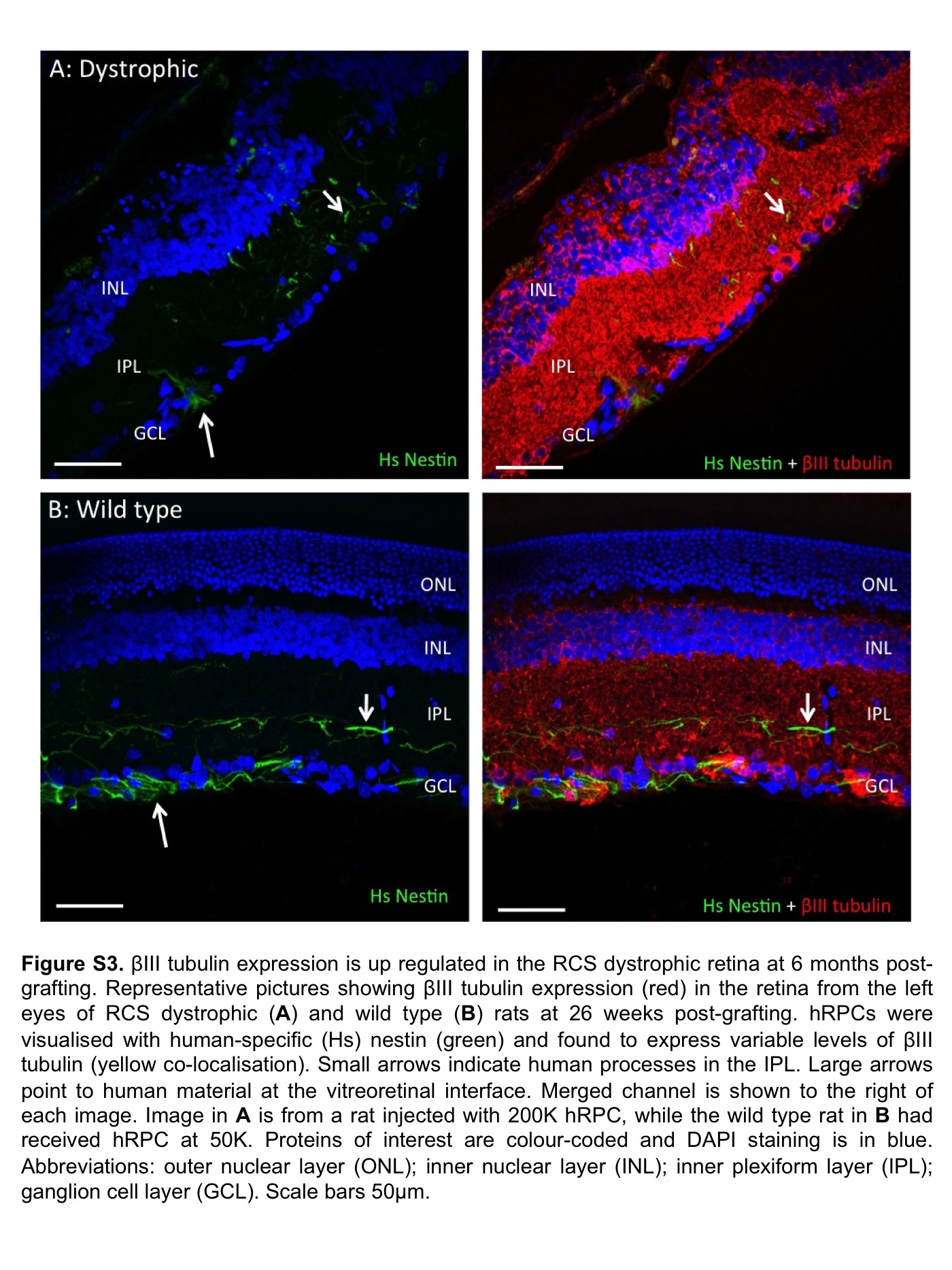

Supplement: Supplement 3 [file i2164-2591-5-4-6-s03.jpg]

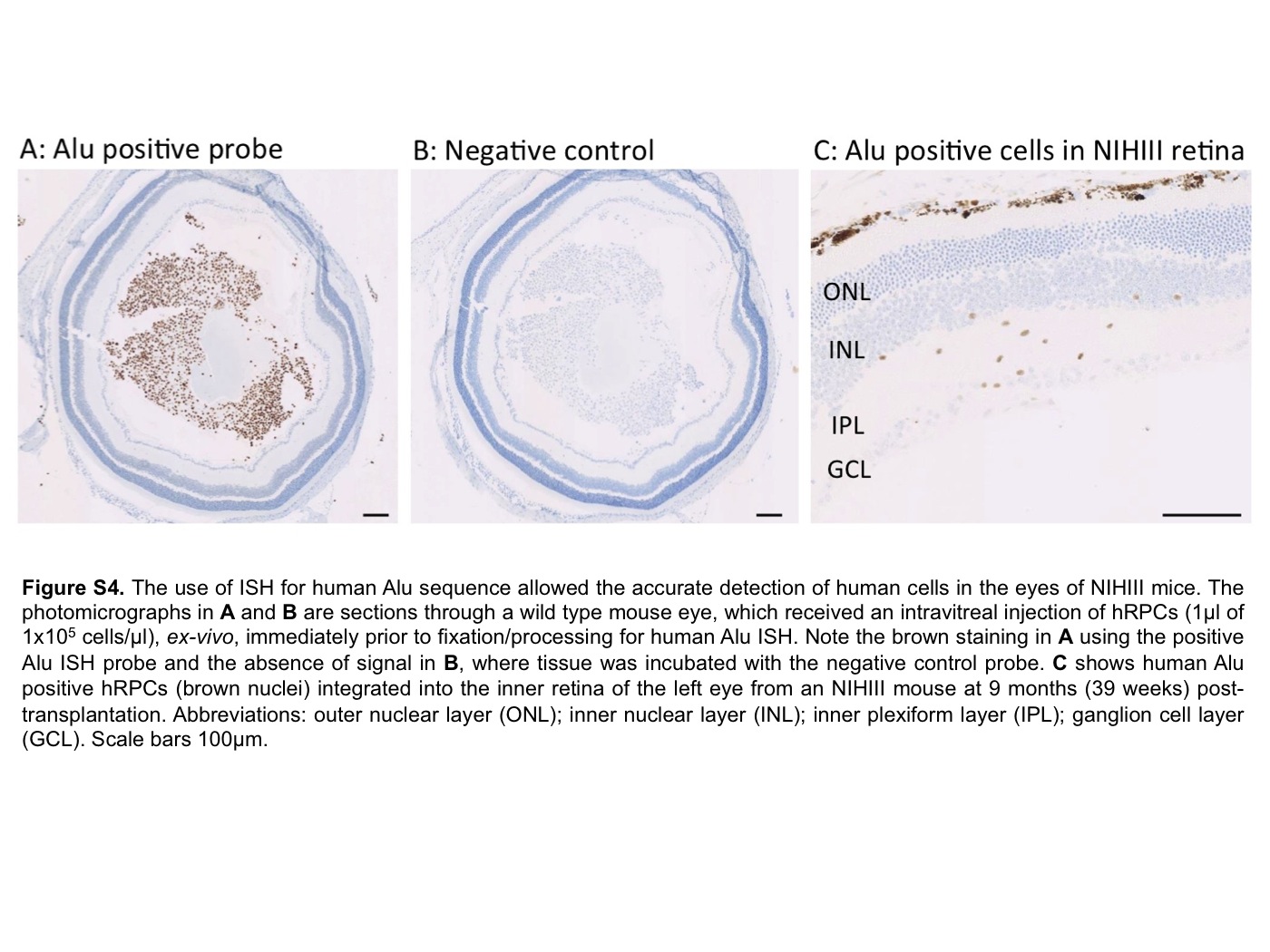

Supplement: Supplement 4 [file i2164-2591-5-4-6-s04.jpg]
