# Supplementary material for: Efficacy and Safety of Human Retinal Progenitor Cells
Source: Transl Vis Sci Technol. 2016 Jul 19;5(4):6. doi: 10.1167/tvst.5.4.6 (PMC4959814; doi:10.1167/tvst.5.4.6)
Supplement: Supplement 5 [file i2164-2591-5-4-6-s05.pdf]

SUPPLEMENTARY METHODS

Subretinal transplantation

Rats were individually weighed and anesthetised by intraperitoneal (i.p.) injection of a ketamine (30mg/kg) / domitor (0.2mg/kg) mixture. The left pupil was then dilated with topical mydriatic drugs and a subretinal injection of hRPC (2µl total volume for rats and 1µl for NIH-III mice) was delivered to the dorsal hemisphere of the left eye using a Leica Wild operating microscope and a custom built Hamilton needle (30 gauge for dystrophic rats, 32 gauge for wild type rats and 34 gauge for NIH-III mice) attached to a 2.5µl Hamilton syringe. In order to prevent hRPC reflux, the subretinal needle was then held in position while a counter-punch was delivered to the lateral aspect of the cornea using a separate, sterile needle (30g). The subretinal injection needle was then retracted to create a self-sealing exit tunnel and both the operated (left) and un-operated (right) eyes were covered with viscotears throughout the surgical procedure and recovery period in order to prevent corneal desiccation. Animals were recovered using Antisedan (6.8mg/kg) and returned to their home cages.

Measurement of the optokinetic response

Rats were habituated to the apparatus before recording data and were tested using the same protocol at either 12 weeks post-transplantation (single dose experiment) or at 12 and 26 weeks post-transplantation (dose ranging study). Every rat was tested 3 times at each of three grating thicknesses: Coarse (0.125 cycles/degree (C/D)), intermediate (0.25 C/D) and fine (0.5 C/D). Rats were placed into a stationary glass internal cylinder surrounded by an external drum illuminated at

8.1-8.2 Ev and lined with a randomly assigned grating. The drum was rotated at a constant speed (12 deg/sec) for 2 periods of 60 seconds in both the clockwise (testing left eye function) and anticlockwise (testing right eye function) direction (total of 4 min of testing). In between each testing period, the drum remained static for 30 seconds. The three different spatial frequencies were presented in different sessions, with no more than two sessions per day. The behaviour of rats was recorded with a digital camera and time spent tracking to the different gratings was then assessed by analysis of behavioural videos. Only those bouts of tracking lasting at least 1 sec were recorded as tracking behaviour.
