# Supplementary material for: Efficacy and Safety of Human Retinal Progenitor Cells
Source: Transl Vis Sci Technol. 2016 Jul 19;5(4):6. doi: 10.1167/tvst.5.4.6 (PMC4959814; doi:10.1167/tvst.5.4.6)
Supplement: Supplement 6 [file i2164-2591-5-4-6-s06.pdf]

SUPPLEMENTARY TABLES

Tissue and regions examined	Necropsy		Histology	Pathology
	Weigh	Fix		Light microscopy
Abnormalities (including masses)		*	*	*
Adrenals		*	*	*
Aorta - thoracic		*	*	*
Bone marrow smear		*		
Brain (cerebellum, cerebrum, midbrain)	*	*	*	*
Caecum		*	*	*
Colon		*	*	*
Duodenum		*	*	*
Epididymides	*	*	*	*
Eyes (left and right), to include optic nerves and harderian gland	*	*	*	*
Femur (femorotibial joint)		b)	*	*
Gall bladder		*	*	*
Head		a)	#	#
Heart (including auricular and ventricular regions)	*	*	*	*
Ileum		*	*	*
Jejunum		*	*	*
Kidneys	*	*	*	*
Liver (section from two lobes)	*	*	*	*
Lungs (section from two major lobes including bronchi)		*	*	*
Lymph nodes - mesenteric		*	*	*
- left axillary		*	*	*
- mandibular		*	*	*
Oesophagus		*	*	*
Ovaries	*	*	*	*
Pancreas		*	*	*
Pituitary		*	*	*
Prostate	*	*	*	*
Salivary glands - submandibular		*	†	†
- parotid		*	†	†
- sublingual		*	†	†
Sciatic nerves		*	†	†
Seminal vesicles		*	*	*
Skeletal muscle		*	†	†
Skin with mammary glands (inguinal area)		*	*	*
Spinal cord (transverse and longitudinal sections at the cervical level)		*	*	*
Spleen	*	*	*	*
Sternum		*	*	*
Stomach		*	*	*
Testes	*	*	*	*
Thyroid with parathyroids		*	*	*
Trachea		*	*	*
Urinary bladder		*	*	*
Uterus with cervix	*	*	*	*
Vagina		*	*	*

In addition, the carcass was retained.

a) Including nasal cavity, paranasal sinuses and nasopharynx.
b) Both hindlimbs retained, one sectioned where appropriate.
* Organs weighed, samples fixed or sections examined microscopically.
# Examined if effects suspected during the study.
† Only one examined.

Table S1. List of tissues that were examined for each NIHIII mouse in the GLP tumorigenicity study.

Tissue
Left Eye
Right Eye
Brain:<br>Cerebrum, Mid brain
Cerebellum,<br>Spinal cord – cervical: LS/TS
Heart:<br>Auricle and Ventricle<br>Spleen
Lung:<br>Left lobe - LS flat,<br>Right Caudal Lobe -TS
Liver x2<br>Pancreas
Mandibular Lymph Node (LN)
Kidney x2

Table S2. Summary of tissues stained by human Alu sequence ISH.

Reagent	Supplier	Product Code	Application / Comment
**EZ Prep**	Roche	950-102	Deparaffinization
**LCS (Liquid Coverslip)**	Roche	650-010	Liquid Coverslip
**RiboWash**	Roche	760-105	Hybridisation Buffer, Stringency Wash, Negative Buffer Control
**Reaction Buffer**	Roche	950-300	Reaction / Wash Buffer
**Alu Positive Control Probe**	Roche	800-2845	Label Alu Repeat Sequence in Human Stem Cells
**Negative Control Probe**	Roche	800-2847	Negative Control Probe – discontinued due to manufacture/supply issues
**Negative (Buffer) Control (RiboWash)**	Roche	760-105	Negative buffer control replacing Negative Control Probe
**Rabbit anti-FITC**	Bio-Rad	4510-7804	Secondary Linking Antibody
**Discovery Ab Diluent**	Roche	760-108	Diluent for Secondary Antibody (Rabbit anti-FITC)
**OmniMap anti Rabbit HRP Multimer**	Roche	760-4311	Detection & Visualisation
**ChromoMap DAB Kit comprising:<br>Inhibitor CM<br>DAB CM, H2O2 CM & Copper CM**	Roche	760-159	Endogenous Peroxidase Block. Chromogen
**Hematoxylin II**	Roche	790-2208	Nuclear Counterstain
**Blueing Reagent**	Roche	760-2037	Blueing of Haematoxylin

Table S3. Reagents used for the human Alu sequence in situ hybridization (ISH) experiment.

Ventana Discovery Ultra Protocol Summary	
1	Deparaffinization [Selected]
2	Warmup Slide to [65 Deg C], and Incubate for [4 Minutes] (Cycle 1)
3	Incubate for [4 Minutes] (Cycle 2)
4	Incubate for [4 Minutes] (Cycle 3)
5	ISH [Selected]
6	1st Probe [Selected]
7	Apply One Drop of **[Appropriate Probe: Alu Positive or Negative Control]** (Probe #1), No Coverslip and Incubate for 8 Minutes
8	Warmup Slide to [85 Deg C], and Incubate for [8 Minutes] (Denaturation)
9	Warmup Slide to [47 Deg C], and Incubate for [1 Hour] (Hybridization)
10	1st Stringency Wash [Selected]
11	Rinse With User Wash #1 RiboWash]
12	Warmup Slide to [45 Deg C], and Incubate for [4 Minutes] (Stringency Wash #1)
13	2nd Stringency Wash [Selected]
14	Rinse With User Wash #2 [RiboWash]
15	Warmup Slide to [45 Deg C], and Incubate for [4 Minutes] (Stringency Wash #2)
16	Linking Antibody [Selected]
17	2nd Antibody [Selected]
18	Warmup Slide to [37 Deg C] from Very Low Temperatures (2nd Antibody)
19	Apply One Drop of [DETECTION 1] (Detection #1), and Incubate for [0 Hr 32 Min]
20	Discovery Detection [Selected]
21	Incubate for [12 Minutes] (Inhibitor CM)
22	HRP Detection [Selected]
23	Apply One Drop of [OMap anti-Rb HRP] (Multimer HRP), and Incubate for [12 Minutes]
24	DAB [Selected]
25	Counterstain [Selected]
26	Use RB for Counterstain [Selected]
27	Apply One Drop of [HEMATOXYLIN II] (Counterstain), and Incubate for [4 Minutes]
28	Post Counterstain [Selected]
29	Use RB for Post Counterstain [Selected]
30	Apply One Drop of [BLUING REAGENT] (Post Counterstain), and Incubate for [4 Minutes]

Table S4. Protocol summary for the human Alu sequence ISH screening experiment.

Procedure	Conditions	Applicable Reagents	Protocol Summary Steps
**Deparaffinization**	3x 4 minute cycles at 65°C	EZ Prep	1 - 4
**ISH Probe**	One drop of appropriate probe / negative control from dispenser. Incubate for 8 minutes	Alu Positive Control Probe<br>Negative Control Probe<br>Negative (Buffer) Control	5 - 7
**ISH Denaturation**	85°C for 8 minutes	N/A	8
**ISH Hybridisation**	47°C for 1 hour	N/A	9
**Stringency Wash**	2x 4 minute Stringency washes at 45°C	RiboWash	10 - 15
**Secondary Link Antibody**	Rabbit anti FITC at 2.0μg/ml, 32 minute incubation at 37°C	Rabbit anti-FITC at 2.0μg/ml Prepared in Discovery Ab Diluent	16 - 19
**Endogenous Peroxide Block**	12 minute incubation	Inhibitor CM– part of ChromoMap DAB Kit	20 - 21
**HRP Detection**	12 minute incubation	OmniMap anti Rabbit	22 - 23
**Chromogen**	ChromoMap DAB Kit	ChromoMap DAB Kit	24
**Counterstain**	4 minute incubation	Haematoxylin II	25 - 27
**Blueing**	4 minute incubation	Blueing Reagent	28 - 30

Table S5. Specific conditions used for the human Alu sequence ISH screening experiment.
